# Genetic mapping of Foxb1-cell lineage shows migration from caudal diencephalon to telencephalon and lateral hypothalamus

**DOI:** 10.1111/j.1460-9568.2008.06503.x

**Published:** 2008-11

**Authors:** Tianyu Zhao, Nora Szabó, Jun Ma, Lingfei Luo, Xunlei Zhou, Gonzalo Alvarez-Bolado

**Affiliations:** 1 Department of Genes and Behavior, Brain Development Group, Max Planck Institute of Biophysical Chemistry37077 Göttingen, Germany; 2 Department of Molecular Developmental Biology, School of Life Sciences, Southwest UniversityChongqing, China

**Keywords:** axon-dependent, forkhead genes, prethalamus, tangential, thalamus

## Abstract

The hypothalamus is a brain region with vital functions, and alterations in its development can cause human disease. However, we still do not have a complete description of how this complex structure is put together during embryonic and early postnatal stages. Radially oriented, outside-in migration of cells is prevalent in the developing hypothalamus. In spite of this, cell contingents from outside the hypothalamus as well as tangential hypothalamic migrations also have an important role. Here we study migrations in the hypothalamic primordium by genetically labeling the *Foxb1* diencephalic lineage. *Foxb1* is a transcription factor gene expressed in the neuroepithelium of the developing neural tube with a rostral expression boundary between caudal and rostral diencephalon, and therefore appropriate for marking migrations from caudal levels into the hypothalamus. We have found a large, longitudinally oriented migration stream apparently originating in the thalamic region and following an axonal bundle to end in the anterior portion of the lateral hypothalamic area. Additionally, we have mapped a specific expansion of the neuroepithelium into the rostral diencephalon. The expanded neuroepithelium generates abundant neurons for the medial hypothalamus at the tuberal level. Finally, we have uncovered novel diencephalon-to-telencephalon migrations into septum, piriform cortex and amygdala.

## Introduction

**Fig. 1:**
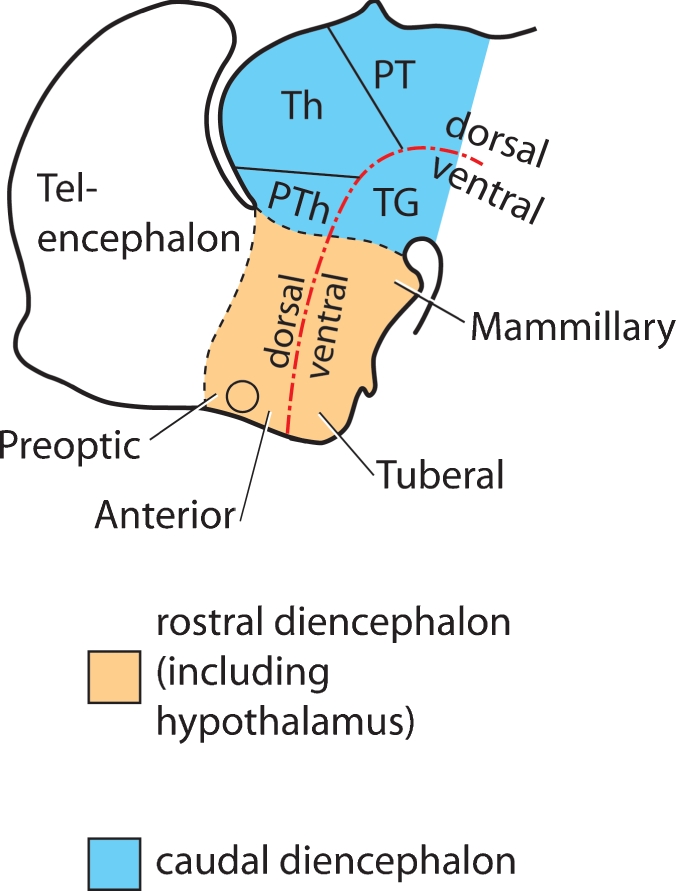
Subdivisions of the diencephalon in the E12.5 mouse embryo.

The hypothalamus is a brain region subserving vital functions, and alterations in its development can cause disease. Obtaining a complete description of how this complex structure is put together during embryonic and early postnatal stages will be helpful in understanding human pathological conditions (Michaud, 2001; Caqueret *et al.*, 2005). The hypothalamus originates in the rostral diencephalon which, because of its situation between telencephalon and caudal diencephalon (including the thalamic region; Fig. 1), undergoes particularly complex patterning (Puelles & Rubenstein, 2003). The longitudinal axis of the neural tube divides the primordium into dorsal and ventral portions (Shimamura *et al.*, 1995). The hypothalamus is subdivided into four areas (preoptic, anterior, tuberal and mammillary; Swanson, 1987; Simerly, 2004), of which the first two are dorsal and the last two are ventral (according to embryonic topology; Fig. 1), although they appear arranged rostrocaudally in the adult brain.

Birthdating shows that most of the hypothalamus develops by waves of neurogenesis from the rostral diencephalic neuroepithelium, followed by radially oriented, outside-in migration (Altman & Bayer, 1986), and gene expression studies confirm this general pattern (Alvarez-Bolado *et al.*, 1995; Caqueret *et al.*, 2006). However, cells from outside the hypothalamus (Muske & Moore, 1988; Schwanzel-Fukuda & Pfaff, 1989; Wray *et al.*, 1989; Henderson *et al.*, 1999) as well as tangential intrahypothalamic migrations (Alvarez-Bolado *et al.*, 2000a) also have an important role. In addition, differential control of migration underlies important functional features such as the sexual dimorphism of some hypothalamic structures (Tobet, 2002). Neuroepithelial expansion is another mechanism resulting in increased cellular heterogeneity in brain regions. As the embryo grows, the neuroepithelium expands by symmetric (horizontal) mitosis (Rakic, 1988; Chenn & McConnell, 1995). Differential expansion of neuroepithelial subpopulations could contribute to regionalization (Alvarez-Bolado *et al.*, 1995). Alternatively, neuroepithelial cells can simply migrate inside the neuroepithelium (Fishell *et al.*, 1993; Arnold-Aldea & Cepko, 1996; Golden & Cepko, 1996). The displaced neuroepithelial cells will generate cells for the region in which they settle.

Ultimately, every forebrain region including the hypothalamus is a composite of cells from different origins (Marin & Rubenstein, 2003). Knowledge of the cell migrations involved is necessary for understanding forebrain development.

Genetic neuroanatomy (Joyner & Zervas, 2006; Dymecki & Kim, 2007) is being successfully used to unravel the development of complex brain regions such as the cerebellum (Zervas *et al.*, 2005; Sillitoe & Joyner, 2007). Similar approaches will be very useful for working out the different cell migrations and lineages in the hypothalamus. Here we have labeled the *Foxb1* diencephalic lineage by crossing a *Foxb1-Cre* mouse line (Zhao *et al.*, 2007) with reporter mouse lines. *Foxb1* is a transcription factor gene widely expressed in the neural tube, with a rostral expression boundary between caudal and rostral diencephalon (Kaestner *et al.*, 1996; Wehr *et al.*, 1997; Alvarez-Bolado *et al.*, 1999, 2000a) which makes this gene useful for studying cell migration into the hypothalamic primordium.

## Materials and methods

### Mouse lines

All experiments with animals were carried out in accordance with the European Communities Council Directive of 24 November 1986 (86/609/EEC) and under authorization Az 32.22/Vo (Ordnungsamt der Stadt Göttingen).

In the *Foxb1*^*Cre*^ mouse line (Zhao *et al.*, 2007; kept in the C57BL/6 background), the *Foxb1* coding sequence was replaced by the Cre recombinase cDNA by homologous recombination. It expresses Cre under the control of the regulatory sequences of *Foxb1*. To characterize the Cre activity encoded by *Foxb1*^*Cre*^, we crossed our mice with the ROSA26R (Soriano, 1999) or Z/AP (Lobe *et al.*, 1999) reporter mouse lines (C57BL/6). All mice used for lineage-labeling were heterozygous for *Foxb1*^*Cre*^ and therefore they were heterozygous for *Foxb1*. *Foxb1* heterozygotes show normal phenotype (Labosky *et al.*, 1997; Wehr *et al.*, 1997; Alvarez-Bolado *et al.*, 2000b; Kloetzli *et al.*, 2001). No homozygotes were used in this study. To obtain embryos, timed-pregnant females of the appropriate crossings were killed by cervical dislocation.

### Lineage labeling

In the *ROSA26R* animals, the reporter gene β-galactosidase was inserted in the constitutively active ROSA locus downstream of a floxed stop codon. Upon Cre-mediated recombination, the stop codon was deleted and β-galactosidase was constitutively produced. Therefore, this reporter is a lineage marker: in mice carrying both the *Foxb1*^*Cre*^ and the ROSA26R alleles, cells expressing *Foxb1* and any cell derived from them will permanently express β-galactosidase. Similar principles apply to the use of Z/AP mice, which carry human placental alkaline phosphatase (hPLAP) as reporter. As hPLAP attaches to axonal membranes it is a very good marker of axons of lineage-labeled neurons (Fields-Berry *et al.*, 1992; Gustincich *et al.*, 1997; Leighton *et al.*, 2001).

The expression of lineage markers is ‘cumulative’ (Louvi *et al.*, 2007), as the lineage domain enlarges as progressively more cells start expressing the marker (here *Foxb1*). In addition, when lineage labeling appears beyond the expression domain of the marker it is indicative of migration.

**Fig. 6:**
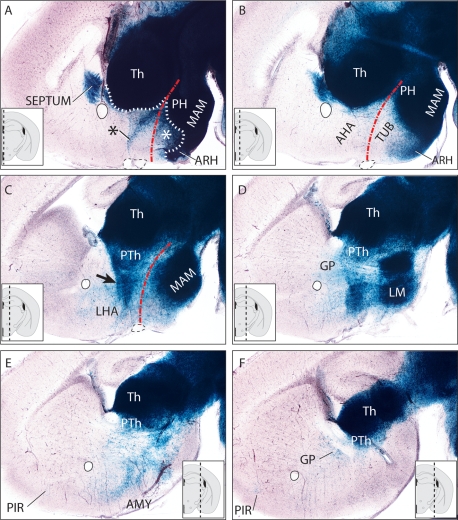
Mantle and neuroepithelium from caudal diencephalon extended into telencephalon and rostral diencephalon. (A–F) Sagittal sections through P0 *Foxb1*^*Cre*^*-ROSA26R* mouse brain stained for β-galactosidase activity. Insets show plane of section. White dotted line in A shows rostral boundary of *Foxb1* lineage at E11.5 (peak expression). Red line in A–C marks approximate dorsal–ventral boundary. Black dotted line in A–C marks (A) the optic chiasm or (B and C) optic tract. (A, B) Medial sections show migration from thalamus into posterior septum (A, B), as well as rostral expansion of the neuroepithelium (asterisks in A). (C) A stream of cells (arrow) links the caudal diencephalic mantle to the lateral hypothalamus at anterior levels. (D) Derivatives of the mammillary area belong to the *Foxb1* lineage. (E, F) Lateral sections show diencephalic migration into telencephalic regions: globus pallidus, ventral pallidum, amygdala and piriform cortex.

We analyzed the *Foxb1* lineage in *ROSA26* crossings at the following ages: embryonic day (E)8.75, E9.5, E11.5, E12.5, E14.5, E15.5, E18.5 and postnatal day (P)0; in *Z/AP* crossings the ages were E12.5, E13.5 and P0. For every embryonic age up to E12.5 we examined two to five litters, and from E15.5 on we examined two to four brains for each age. Both Figs 6 and 7 show material from one brain, for consistency. We identified structures in the brain according to current reference works (Alvarez-Bolado & Swanson, 1996; Paxinos & Franklin, 2001).

### In situ hybridization (ISH)

Whole-mount ISH was performed as described (Wilkinson, 1992). The *Foxb1* probe was cloned by PCR (forward primer atc gct agg gag tac aag atg cc; reverse gat cag tga gtt ggt ctt gtg gc). Briefly, the embryos were fixed in 4% formaldehyde in phosphate-buffered saline (PBS) overnight at 4°C, washed in PBS with 0.1%Tween-20 (PBT) and stored at −20°C in methanol. For ISH the embryos were rehydrated, bleached in 6% H_2_O_2_, digested in 10 μg/mL Proteinase K in PBT at room temperature (RT), washed in 2 mg/mL glycine in PBT, postfixed in 4% PFA and 0.2% glutaraldehyde in PBT, prehybridized for 1–2 h at 70°C and hybridized overnight at 70°C. They were then washed in 50% formamide, 5× SSC, pH 4.5, and 1% SDS at 70°C, rinsed in 100 mm maleic acid, 150 mm NaCl, 2 mm levamisole and 0.1% Tween-20 (MAB) and incubated in 10% sheep serum in MAB with 2% Blocking Reagent (Roche Diagnostics GmbH, Mannheim, Germany) for 2–3 h at RT, then in anti-DIG AP antibody (Roche) overnight at 4°C. The embryos were rinsed, then left in MAB overnight at 4°C. They were then incubated in BM-Purple (Roche) with levamisole at RT and, after color developed, washed in PBT (pH 4.5), fixed in 4% formaldehyde with 0.1% glutaraldehyde overnight at 4°C, and transferred into 80% glycerol in PBT.

### β-Galactosidase activity detection

β-Galactosidase activity was detected as described (Koenen *et al.*, 1982). Embryos from timed pregnancies were collected, washed with cold PBS and fixed for 30–50 min in 1% paraformaldehyde, 0.2% glutaraldehyde and 0.02% NP40 in PBS. The embryos were then rinsed and incubated in staining solution [1 mg/mL Xgal, 2 mm MgCl2, 5 mm K3Fe(CN)6 and 5 mm K4Fe(CN)6) in PBS] overnight in the dark at RT. For animals older than E12.5, the brains were dissected out, fixed in 4% paraformaldehyde for 60 min at 4°C, embedded in agarose and cut into sections (150 μm). The sections were fixed on ice for 30 min, washed with PBS, incubated with staining solution and fixed again in 4% paraformaldehyde for 60 min.

### Alkaline phosphatase activity detection

Material was collected, fixed in 4% paraformaldehyde on ice for 60 min, embedded in agarose and cut into sections (150 μm). The sections were fixed again in 4% paraformaldehyde with 0.2% glutaraldehyde on ice for 60 min, rinsed, incubated for 30 min at 72°C to inhibit endogenous phosphatase activity, rinsed in alkaline phosphatase buffer (100 mm Tris–Cl, pH 9.5, 100 mm NaCl and 10 mm MgCl_2_), incubated with staining solution (250 μL nitro-blue-tetrazolium and 187.5 μL 5-bromo-4-chloro-3-indolyl phosphate per 50 mL in alkaline phosphatase buffer) overnight at 4°C, and fixed in 4% paraformaldehyde for 60 min at 4°C (Lobe *et al.*, 1999).

### Immunohistochemistry

Paraffin sections (15 μm) of P0 *Foxb1*^*Cre*^*/ROSA26R* mouse brains were dewaxed, preincubated in PBT with 10% fetal calf serum and incubated overnight at 4°C in antibody. Alexa (Molecular Probes-Invitrogen, Karlsruhe, Germany) fluorescent secondary antibodies were used for visualization (1 : 500). Antibodies were: anti-MCH (rabbit, 1 : 100), Phoenix Pharmaceuticals, Burlingame, CA, USA; anti-orexin (mouse, 1 : 10), R&D Systems GmbH, Wiesbaden, Germany; anti-GAD ‘pan’ antibody (rabbit, 1 : 100), Abcam, Cambridge, UK; anti-calbindin (rabbit, 1 : 100), Chemicon-Millipore GmbH, Schwalbach, Germany; anti-calretinin (rabbit, 1 : 200), Swant, Bellinzona, Switzerland; anti-β-galactosidase (chicken, 1 : 100), Abcam; anti-glial fibrillary acidic protein (GFAP; rabbit, 1 : 200), DakoCytomation; anti-neurofilaments (2H3, 1 : 5; this mouse monoclonal antibody, developed by T. M. Jessell and J. Dodd, was obtained from the Developmental Studies Hybridoma Bank developed under the auspices of the NICHD and maintained by The University of Iowa, Department of Biology, Iowa City, IA, USA).

### Microscopy

Leica DMR and MZ APO microscopes (Leica Mikrosysteme, Wetzlar, Germany), Olympus DP50 cameras (Olympus, Tokyo, Japan) and Cell-F 2.6 software (Olympus Soft Imaging Solutions GmbH, Münster, Germany) were used for analysis and photography. Image contrast was enhanced by applying Photoshop 7.0 (Adobe Systems Inc., San José, CA, USA) software tools to one whole image file at a time.

## Results

### Lineage-labeling with the *Foxb1-Cre* mouse line

The *Foxb1-Cre* mouse line (Zhao *et al.*, 2007) carries the recombinase *Cre* under the control of the regulatory sequences of *Foxb1*. To use this line as a marker of the *Foxb1* lineage, we crossed it with reporter mouse lines engineered to carry inactive reporter genes (e.g. easily detected enzymes such as β-galactosidase or alkaline phosphatase) in every cell. In the mouse progeny that we obtained upon crossing the *Foxb1-Cre* mouse line with a reporter mouse line, every cell expressing *Foxb1* carried a reporter gene made permanently active by the Cre recombinase. As the active reporter gene is inherited by any cells derived from *Foxb1*-expressing neuroepithelium, the full *Foxb1* lineage was labeled (see Materials and methods). The mice showed otherwise normal appearance and behavior.

**Fig. 2:**
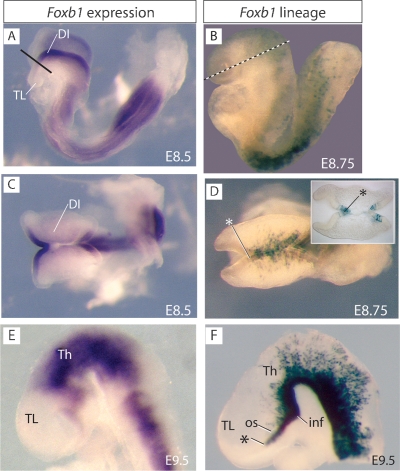
Transient expression but extended lineage of *Foxb1* in the diencephalon. (A) *Foxb1* whole-mount ISH on E8.5 mouse embryo showing expression in diencephalon, not telencephalon (separated by a straight line). (B) β-galactosidase detection on *Foxb1*^*Cre*^*-ROSA26R* embryo at the beginning of neurulation. (C) shows the same embryo as in (A) from the top. (D) View from top of the embryo in B showing the basal plate of the presumptive diencephalon. Inset shows section through the dotted line in B. *Foxb1*-expressing cells are found in the diencephalic ventral midline and adjacent to it. Asterisks mark the rostral tip of expression in the ventral midline of D and inset. (E, F) At E9.5, *Foxb1* mRNA had disappeared from the ventral rostral diencephalon (E), while lineage labeling shows the ventral midline labeled up to eye level (asterisk in F). For abbreviations in this and subsequent Figures see main list.

**Fig. 3:**
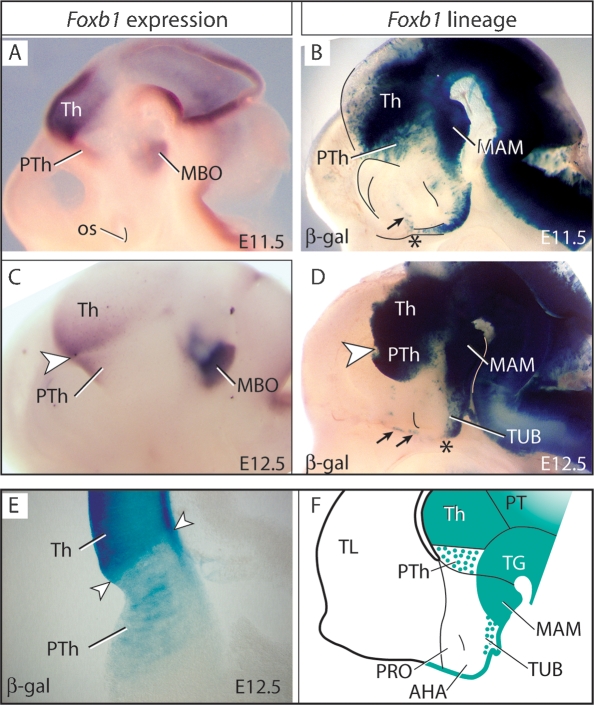
*Foxb1* expression in the caudal diencephalon peaked early and disappeared. (A, C) *Foxb1* whole-mount ISH on hemisected embryonic mouse heads. (B, D) β-Galactosidase activity detection on hemisected embryonic *Foxb1*^*Cre*^*-ROSA26R* mouse heads. (A) At E11.5, *Foxb1* expression peaked in thalamus, prethalamus and midbrain. Rostral to the mammillary body there was no expression. (B) *Foxb1* lineage labeling at E11.5 occupied the ventral midline of the entire neural tube with a rostral limit at the level of the lamina terminalis (asterisk). Arrow points at *Foxb1*-lineage cells in the basal ganglia region. The thalamus, prethalamus, mammillary area and part of the tuberal area are also labeled. (C) At E12.5, *Foxb1* expression in the caudal diencephalon was disappearing. White arrowhead, zona limitans. (D) At E12.5, the *Foxb1* lineage formed most of the dorsal and ventral caudal diencephalon, including part of the prethalamus (ventral thalamus). The mammillary and part of the tuberal areas are labeled. White arrowhead, zona limitans. Asterisk marks rostralmost extent of *Foxb1* lineage; scattered cells were found more dorsally (arrows). (E) Transverse section through the caudal diencephalon of an E11.5 embryo. Arrowheads, zona limitans. (F) Diagram summarizing the distribution of *Foxb1*-lineage cells in the diencephalon at E12.5.

To identify cell migration by lineage labeling, we compared the location of cells expressing *Foxb1* with the location of *Foxb1*-lineage cells: of necessity, any lineage-labeled cells found beyond the *Foxb1* expression boundary had either migrated from the *Foxb1*-expressing domain or were derived from mitotic neuroepithelium that had expanded from the *Foxb1*-expressing domain. For this reason, we first recorded the expression domains of *Foxb1* in the early diencephalon (Figs 2 and 3), using the nomenclature summarized in Fig. 1 (Puelles & Rubenstein, 2003).

### Transient expression but extended lineage of Foxb1 in the diencephalon

Aspects of the *Foxb1* expression pattern have been reported, particularly at late prenatal–postnatal stages (Kaestner *et al.*, 1996; Alvarez-Bolado *et al.*, 1999). For our lineage analysis we needed a complete and systematic exploration of the dynamic changes in the rostral expression boundary, not yet available in the literature.

*Foxb1* is expressed in the neural plate from diencephalon to spinal cord (Fig. 2A; also Ang *et al.*, 1993; Zhao *et al.*, 2007). Early *Foxb1-Cre/ROSA26R* embryos stained *in toto* for β-galactosidase activity showed a general distribution of labeling similar to that of *Foxb1* mRNA (Fig. 2B). Relatively weak and transient expression in the early diencephalon makes it difficult to assess the distribution of *Foxb1* mRNA in the open neural tube (Fig. 2C). Lineage labeling, however, yielded a clear picture, although it appeared with a short time lag after *Foxb1* expression (after the *Foxb1* locus becomes active, the Cre recombinase has to be produced at high enough levels to render the reporter gene active, and the reporter enzyme has to be synthesized). Careful examination revealed *Foxb1* lineage-labeled cells in the ventral midline of the presumptive diencephalon and midbrain (Fig. 2D), as was confirmed by sectioning (inset in Fig. 2D).

At E9.5, *Foxb1* expression was disappearing from the ventral side of the diencephalon and the ventral midline was no longer labeled (Fig. 2E). Lineage labeling showed a faithful record of previous *Foxb1* expression in the ventral midline (cumulative labeling; Louvi *et al.*, 2007), which was labeled up to eye levels (asterisk in Fig. 2F). The ventral portion of the diencephalon, including the infundibular region, was labeled as well. At this age, *Foxb1* expression was high in the dorsal portion of the neural tube, including caudal diencephalon (thalamic region) and midbrain (Fig. 2E). Accordingly, lineage labeling began to appear in these regions (Fig. 2F). At E9.5 the telencephalon was free from *Foxb1* expression (Fig. 2E) and *Foxb1*-lineage cells (Fig. 2F).

### Foxb1 expression in the caudal diencephalon peaked early and disappeared

At E11.5, expression of *Foxb1* reached its peak of intensity in the caudal diencephalon, including a large thalamic (dorsal thalamic) domain and a small prethalamic (ventral thalamic) domain (Fig. 3A). Expression in the rostral diencephalon (including the hypothalamic primordium) strongly and specifically labeled the mammillary body (Fig. 3A). Horizontal sections, however, demonstrated that at this stage *Foxb1* was expressed in the mantle but was no longer expressed in the neuroepithelium (not shown). In the caudal diencephalon, thalamus and tegmentum were heavily labeled while the prethalamus showed labeled and unlabeled cells. In the rostral diencephalon, the dorsal portion did not show any *Foxb1*-lineage cells. In the ventral portion, the mammillary area and adjacent regions were labeled, as well as some cells in the tuberal area (Fig. 3B). The *Foxb1* lineage in the ventral midline extended rostrally into the retrochiasmatic portion of the basal plate and reached the level of the lamina terminalis (asterisk in Fig. 3B). At this point, in some cases, the labeled neuroepithelium extended into the basal ganglia (arrow).

At E12.5 the major brain regions are recognizable by specific gene expression (Shimamura *et al.*, 1995). At this age, *Foxb1* expression had almost completely vanished from the caudal diencephalic neuroepithelium, except for very weak domains in the thalamus and prethalamus (Fig. 3C), separated by a clear boundary corresponding to the zona limitans interthalamica (arrowhead in Fig. 3C). In the rostral diencephalon, the mantle layer (postmitotic neurons) of the mammillary body was also very strongly labeled at this age (Fig. 3C). Lineage labeling at E12.5 covered the thalamus as well as part of the prethalamus in the caudal diencephalon (Fig. 3D). The ventral portion of the caudal diencephalon was also completely labeled (Fig. 3D). In the rostral diencephalon, mammillary area labeling covered a domain larger than the actual *Foxb1* expression in the mammillary body. Numerous labeled cells were also present in the tuberal area (Fig. 3D). The labeled ventral midline seemed to have ceased expanding rostrally, and its rostral end was at this stage caudal to eye levels (asterisk in Fig. 3D). However, scattered labeled cells were found at the level of the lamina terminalis (arrows in Fig. 3D).

In transverse sections, the thalamic neuroepithelium was intensely labeled while the prethalamic neuroepithelium showed labeled and unlabeled cells. The two domains were clearly separated by a sharp boundary (zona limitans; arrowheads in Fig. 3E). Figure 3F summarizes our findings.

### Neuroepithelial migration from diencephalon into the early telencephalon

**Fig. 4:**
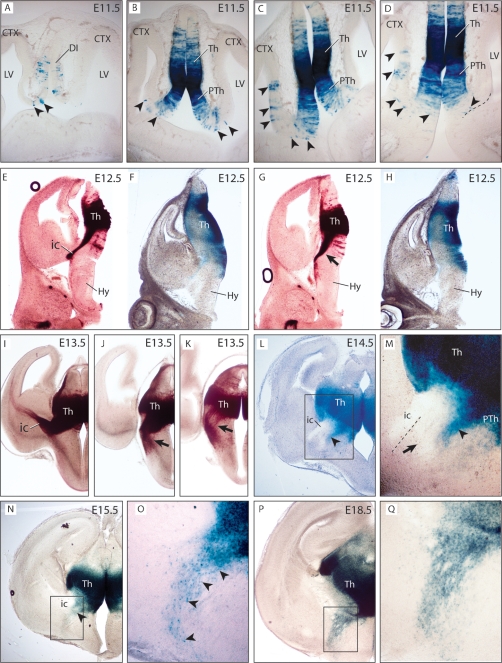
*Foxb1*-lineage cell migration and axonal bundle formation. Reporter detection on transverse sections through (A–D, F, H, L–Q) *Foxb1*^*Cre*^*-ROSA26R* and (E, G, I–K) *Foxb1*^*Cre*^*-Z/AP* brains of the ages indicated. (A–D) E11.5 brains. Arrowheads point at *Foxb1*-lineage labeled cells seemingly abandoning the diencephalon to enter the medial cortex. (A) shows the most rostral, (D) the most caudal section. No labeled cells were found rostral to A or caudal to B. Dotted line in D, interventricular connection. (E–H) E12.5 brains. Rostral sections (E and G) show internal capsule (ic) formation. Caudal sections (G and H) show formation of thalamohypothalamic axonal bundle (arrow in G) before *Foxb1*-lineage cells appeared in the hypothalamus (Hy; H). (I–K) E13.5 brains. Rostral section (I) shows internal capsule; more caudal sections show thalamohypothalamic axons (arrow in J and K). (L, M) E14.5 brains. The area framed in L is shown magnified in M. The internal capsule (ic) can be seen together with a ventrally directed axonal bundle (arrow in M). Foxb1-lineage cells (arrowheads in L and M) start migrating alongside these axons. (N, O) E15.5 brains. The area framed in N is shown magnified in O. Migrating cells (arrowheads) from the prethalamus stream into the hypothalamus. (P, Q) E18.5 brains. The area framed in P is shown magnified in Q. The migratory stream is completely established.

*Foxb1* has been reported as a diencephalic marker, not expressed in the early telencephalon of the mouse or zebrafish (Kaestner *et al.*, 1996; Wehr *et al.*, 1997; Varga *et al.*, 1999). We confirm this (Figs 2E and 3A and C), indicating that any *Foxb1*-lineage cell found in the telencephalon would have migrated from diencephalic levels. As labeled cells could have been overlooked due to the evagination of the telencephalic vesicles after E10.5, we examined lineage-labeled, transversely sectioned E11.5 brains (Fig. 4A–D). Surprisingly, at the level where diencephalon and telencephalon are continuous, a trail of labeled neuroepithelial cells entered the cortex (arrowheads in Fig. 4A–D). The apparent origin of the migrating neuroepithelial cells was in the prethalamus (Fig. 4B–D). No labeled cells were found in the telencephalon at levels rostral or caudal to the ones shown in Fig. 4A or Fig. 4D, respectively.

### Extension of axons into hypothalamus before the beginning of migration

To explore the relation of the *Foxb1* lineage with the early thalamic axons we used the Z/AP reporter mouse line, which carries as reporter an enzyme that attaches to axonal membranes, efficiently labeling the *Foxb1* lineage axons (see Materials and methods). At ∼E12.5, the thalamocortical projection (internal capsule) was visible (Fig. 4E and F). More caudally, we observed an axonal bundle directed ventrally towards the hypothalamus (Fig. 4G). At this age, the region of the hypothalamus receiving the axons was devoid of *Foxb1*-lineage cells (Figs 3C and D and 4F and H). As development proceeded, the thalamocortical and thalamohypothalamic *Foxb1*-lineage bundles elongated and became increasingly distinct (Fig. 4I–K).

### Migratory routes into ventral diencephalon and telencephalon

Mapping the relation between the *Foxb1*-lineage domains and the spatiotemporal pattern of *Foxb1* expression in the forebrain (Figs 2 and 3) until it becomes stable or disappears was the prerequisite to the analysis of cell migration into the hypothalamic primordium. From this point on, detection of *Foxb1* lineage-labeled cells rostral to the boundary would be indicative of migration. At E14.5, *Foxb1*-lineage cells apparently from the prethalamus started migrating in the rostral and ventral direction (arrowheads in Fig. 4L and M). The migrating cells were associated with thalamohypothalamic axons (arrow in Fig. 4M) and not with the internal capsule (ic in Fig. 4L and M). This phenomenon became more clear at E15.5, when the migrating cells reached the ventralmost level of the hypothalamus (Fig. 4N and O). At E18.5 the migrating cell group appeared completely established (Fig. 4P and Q).

**Fig. 5:**
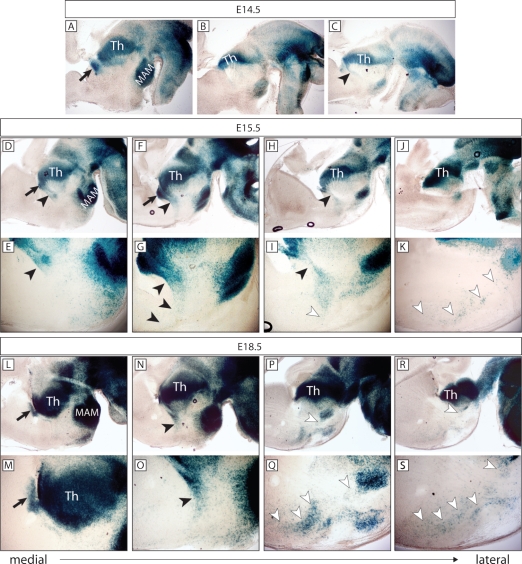
Migration routes of *Foxb1*-lineage cells in the forebrain Reporter detection on sagittal sections (rostral to the left) through *Foxb1*^*Cre*^*-ROSA26R* brains of the ages indicated. (A–C) At E14.5, labeled cells appeared expanding in the rostral direction (arrow in A) as well as leaving the thalamic region in the ventral direction (arrowhead in C). No migrating cells were present at medial levels (A and B) or in the ventral forebrain. (D, F, H and J) E15.5 sagittal sections at low magnification and the corresponding high magnification details (E, G, I and K) show how labeled cells from the thalamic region move towards the septum (arrows in D and F). Other labeled cells streaming from the thalamic region (black arrowheads in D–I) reached ventral levels. At lateral levels, labeled cells from the caudal hypothalamus entered the telencephalon (white arrowheads in I and K). (L, N, P and R) E18.5 sagittal sections at low magnification and the corresponding high magnification details (M, O, Q and S). Arrows in L and M show the labeled septum. Black arrowheads in N and O mark the thalamohypothalamic migration. White arrowheads in P–S mark labeled cells at the boundary between hypothalamus and ventral telencephalon.

On sagittal sections at different ages (Fig. 5A–S) we detected two additional migratory routes. Cells from the prethalamus extended rostrally into the presumptive septum on medial levels from E14.5 (arrow in Fig. 5A) through E15.5 (arrows in Fig. 5D and F) to E18.5 (arrows in Fig. 5L and M). The migration into the hypothalamus (see above, Fig. 4) was also evident on sagittal sections at E14.5 (black arrowhead in Fig. 5C), E15.5 (black arrowheads in Fig. 5F–I) and E18.5 (black arrowheads in Fig. 5N and O). In a third migration route, labeled cells from the caudal hypothalamus migrated into ventral levels of the telencephalon by taking advantage of the ventral diencephalon–telencephalon continuity. This migration became apparent at E15.5 (white arrowheads in Fig. 5I and K) and was very substantial at E18.5 (white arrowheads in Fig. 5P–S).

### Rostral expansion of caudal diencephalic neuroepithelium

**Fig. 7:**
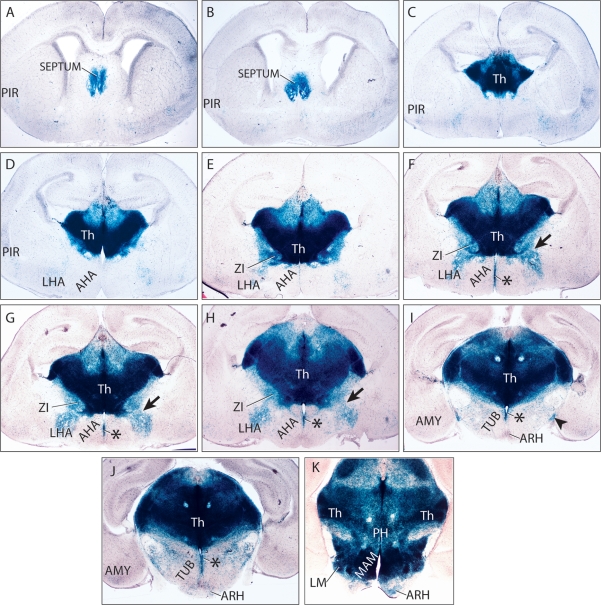
Different regions of the lateral hypothalamus showed different patterns of colonization by *Foxb1*-lineage cells. (A–K) Transverse sections through a P0 *Foxb1*^*Cre*^*-ROSA26R* mouse brain stained for β-galactosidase activity. (A, B) Contribution of *Foxb1* lineage to the telencephalon: septum, pirifom cortex and ventral pallidum. (C) The most rostral portion of the lateral hypothalamus did not receive *Foxb1*-lineage cells. (D–H) The lateral hypothalamus at anterior levels received a stream of cells (arrow in F–H) apparently from the zona incerta. (I, J) At tuberal levels, the medial and lateral hypothalamus had abundant labeled scattered cells presumably originating in the labeled neuroepithelium. Arrowhead in I marks a previously known *Foxb1*-expressing population. (K) The entire mantle of the mammillary area is from *Foxb1* lineage. Asterisk in F–J shows the *Foxb1*-lineage neuroepithelium of the third ventricle.

We went on to map the *Foxb1* lineage onto the hypothalamus and telencephalon at postnatal P0, when regionalization and most mantle formation are over (Figs 6 and 7).

Sagittal sections through the midline showed that the boundary of the most intensely labeled neuroepithelium (white dotted line in Fig. 6A) was broadly similar to that found at earlier stages, immediately after *Foxb1* expression had disappeared from the caudal diencephalon (Fig. 3D). However, we observed several intriguing departures from this pattern, i.e., labeled cells located rostral to the boundary, in keeping with the migrations observed at earlier stages (Figs 4 and 5).

A region of neuroepithelium rostral to the boundary was labeled with β-galactosidase (asterisks in Fig. 6A); this was much more rostrally positioned than any *Foxb1*-expressing cell had been, and than any *Foxb1*-lineage cell that could be seen at E12.5 (compare with Fig. 3D). These cells demonstrate a rostral expansion of the caudal diencephalic neuroepithelium (dorsally) and of the mammillary neuroepithelium (ventrally). Transverse sections confirmed this expansion (asterisks in Fig. 7F–J).

### Migration stream from prethalamic levels into the lateral hypothalamus

In the mantle layer, we observed a striking departure from the early *Foxb1* expression and lineage boundaries. A large group of cells was positioned between the zona incerta (prethalamus or ventral thalamus) and the hypothalamus (arrow in Figs 6C and 7F–H). This cell group seemed the result of the migration stream detected at E14.5 (Figs 4L and N and 5) and it was sharply delimited lateromedially (compare Fig. 6C to B and D) and rostrocaudally (arrows in Fig. 7F–H), ending in the lateral hypothalamus at anterior levels.

### The lateral hypothalamus at the tuberal level

More caudally (tuberal level), the medial and lateral hypothalamus showed abundant scattered *Foxb1*-lineage cells (Fig. 7I and J). These cells must have either migrated caudorostrally through the mantle layer (presumably from the mammillary area) or were generated in the expanded neuroepithelium (asterisks in Figs 6A and 7I and J). A small and compact group of labeled cells was found consistently in the lateral hypothalamus at this level (arrowhead in Fig. 7I).

### The mantle in the mammillary region

We have mentioned the expansion of the labeled mammillary neuroepithelium (see above). In the rostral diencephalic mantle, the entire mammillary body was formed by cells of the *Foxb1* lineage (Figs 6B–D and 7K), in agreement with the *Foxb1* expression pattern (Fig. 3C). The posterior hypothalamus was also formed by expanded mammillary neuroepithelium, as could be expected from the lineage labeling at E12.5 (Fig. 3C and D).

### Diencephalon-to-telencephalon migrations, medial and lateral

We established that the early expression domain of *Foxb1* did not reach the telencephalon (Figs 2–4). However, we found a number of *Foxb1* lineage-labeled cells in the telencephalon at P0. The presence of such labeled cells must have been the result of migration from caudal levels or cell division from migrated neuroepithelium (Fig. 4). At medial levels, labeled cells formed a migration stream into the posterior septum (septofimbrial nucleus and triangular nucleus of the septum) which was clear on sagittal sections (Fig. 6A and B) as well as in transverse sections (Fig. 7A–C). At lateral levels, abundant *Foxb1*-lineage cells migrated from the thalamic region into the amygdala, globus pallidus and piriform cortex (Figs 6E and F and 7A–C).

### Telencephalic settling places of Foxb1-lineage cells

**Fig. 8:**
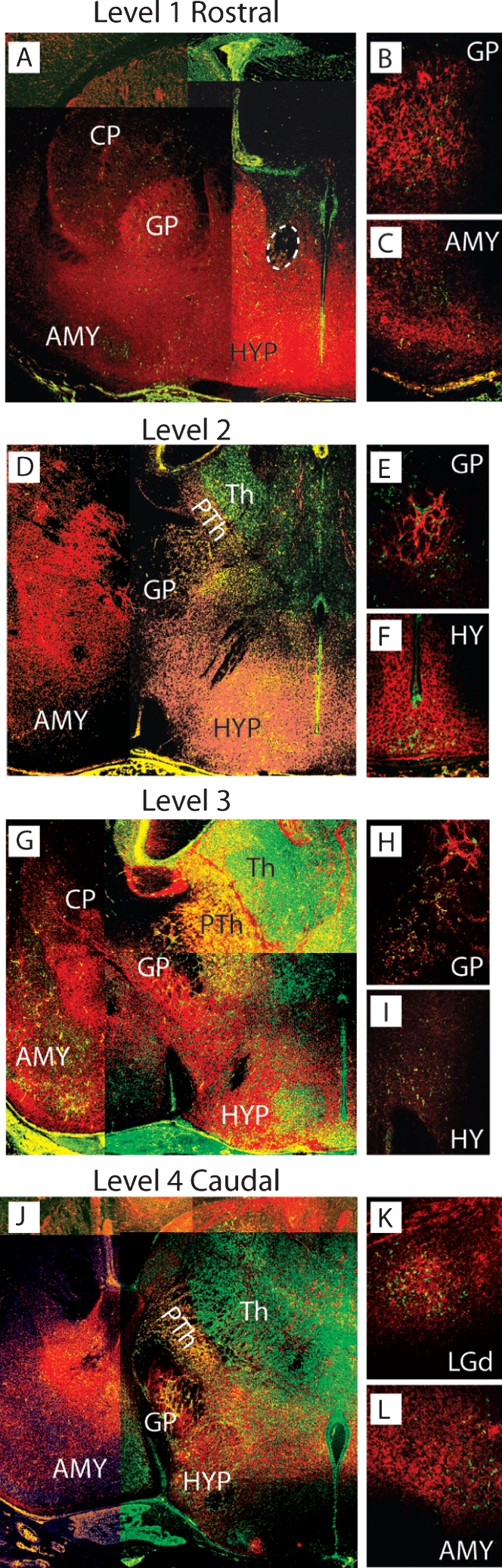
*Foxb1*-lineage cells in basal ganglia and hypothalamus. (A, D, G and J) Transverse sections and (B, C, E, F, H, I, K and L) high-magnification details at four characteristic rostrocaudal levels of a P0 *Foxb1*^*Cre*^*-ROSA26R* brain labeled with antibodies for GAD (red) and β-galactosidase (green). Scattered *Foxb1*-lineage cells were present in the hypothalamus, amygdala and globus pallidus at every level. (K) The thalamus was devoid of GAD (except in the lateral geniculate nucleus) but (D, G and J) showed abundant *Foxb1*-lineage cells. (G, J) The prethalamus showed abundant colocalization of the two markers. Dotted line in A, fornix.

To confirm the identity of the telencephalic regions receiving *Foxb1*-lineage cells, we labeled transverse sections of *Foxb1*^*Cre*^*-ROSA26R* neonatal brains (P0) with antibody against β-galactosidase and against glutamic acid decarboxylase (GAD). The distribution of GAD in cell bodies and axon terminals has a well-known and characteristic regional pattern (see for instance the Allen Brain Atlas: http://mouse.brain-map.org) allowing for the identification of the major areas of the forebrain (Fig. 8). The results confirmed our previous detection of *Foxb1*-lineage cells in the globus pallidus, amygdala and lateral hypothalamus (Figs 5 and 6).

### Characterization of Foxb1-lineage cells in telencephalon and hypothalamus

**Fig. 9:**
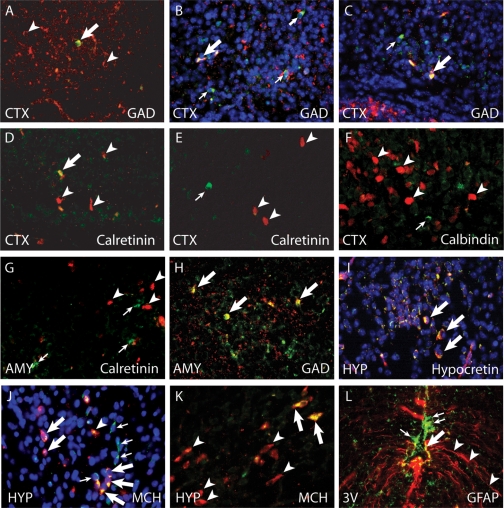
Characterization of *Foxb1*-lineage cells in the forebrain. (A–L) Antibody detection of cell type-specific markers (red; marker as indicated on the panels) and β-galactosidase (green) in (A–F) the cortex, (G and H) amygdala, (I–K) hypothalamus and (L) third ventricle of P0 *Foxb1*^*Cre*^*-ROSA26R* brains. Large arrows mark colocalization, small arrows mark *Foxb1*-lineage cells without marker colocalization, and arrowheads mark cells expressing a specific marker but no β-galactosidase.

We then carried out an initial characterization of *Foxb1*-lineage cells with antibodies against different neuronal and glial markers (Fig. 9). In the cortex, *Foxb1*-lineage cells were not abundant, and most of them expressed GAD (Fig. 9A–C). Calretinin was a less frequent marker (Fig. 9D and E), and we could not detect colocalization with calbindin in any case (Fig. 9F). *Foxb1*-lineage cells in the amygdala colocalized mostly with GAD (Fig. 9G and H). Two very specific markers of cell populations in the lateral hypothalamus are hypocretin (also known as orexin) and melanin concentratin hormone (MCH; see for instance Cvetkovic *et al.*, 2004). Some of the lateral hypothalamic *Foxb1*-lineage cells coexpressed hypocretin/orexin (Fig. 9I) or, more often, MCH (Fig. 9J and K). None of the *Foxb1*-lineage cells coexpressed GFAP, a glial marker. The exception was the lining of the third ventricle, where some radial glial cells (which also express GFAP) were double-labeled (Fig. 9L), in agreement with our detection of *Foxb1* lineage in the hypothalamic neuroepithelium (Fig. 7F–J).

### Axon-dependent migration into the lateral hypothalamus

**Fig. 10:**
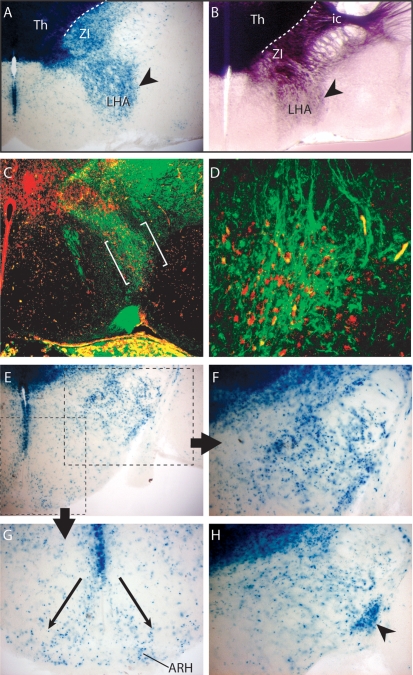
Migration strategies into the hypothalamus. (A, E–H) Transverse sections through a P0 *Foxb1*^*Cre*^*-ROSA26R* brain stained for β-galactosidase activity. (B) Transverse section through a P0 *Foxb1*^*Cre*^*-Z/AP* mouse brain stained for alkaline phosphatase activity. (C) Transverse section through a P0 *Foxb1*^*Cre*^*-ROSA26R* brain labeled for neurofilaments (green) and β-galactosidase (red). (D) High-magnification image of the area framed in C. (A, B) A large and compact group of cells (arrowhead in A) entered the rostral portion of the lateral hypothalamus from the zona incerta. Axons connecting zona incerta and lateral hypothalamus (arrowhead in B) in the same regions as the migrating cells in A. (C, D) Axons from the prethalamus entered the lateral hypothalamus (framed in C). *Foxb1*-lineage cells (red) can be seen among the axons in (D) the high-magnification panel. (E) Labeled hypothalamic neuroepithelium in the tuberal area. The areas framed in E are shown magnified in F and G. (F) Nonradially arranged cells in the lateral hypothalamus (tuberal level). (G) Radially arranged cells in the medial hypothalamus (tuberal level). Arrows indicate direction of migration. (H) Compact group of non-radial labeled cells (arrowhead) in the lateral hypothalamus (tuberal level).

Intriguingly, the migration from prethalamus into the lateral hypothalamus was tangential (rostrocaudal; see Fig. 1), suggesting that axons rather than radial glia could be the substrate (Fig. 10A). In brains from *Foxb1-Cre/ZAP* crossings, we detected an important axonal bundle apparently coursing from the prethalamic region into the lateral hypothalamus (Fig. 10B). The β-galactosidase domain and the alkaline phosphatase domain coincided in size, shape and position (Fig. 10A and B), suggesting the axon-dependent nature of this migration. By using antineurofilament antibody (monoclonal antibody 2H3, green in Fig. 10C and D) and anti-β-galactosidase antibody (red in Fig. 10C and D) we confirmed the existence of this axonal bundle.

### Diverse migration strategies into the tuberal portion of the lateral hypothalamus

**Fig. 11:**
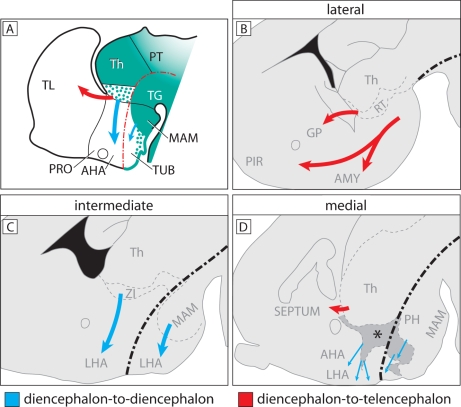
Multiple diencephalon-originating rostral migrations. (A) Our findings summarized on a diagram of the E12.5 diencephalon. (B) Diencephalon-to-telencephalon migrations on the lateral plane. (C) Diencephalon-to-diencephalon migrations in the dorsal and ventral portions. (D) Diencephalon-to-telencephalon migrations in the medial plane (red arrow), and migrations from the rostrally expanded neuroepithelium (blue arrows). Asterisk marks the expanded neuroepithelium.

At tuberal levels, the lateral hypothalamus showed abundant labeled cells. In contrast to the compact arrangement of *Foxb1*-lineage cells at anterior levels (Fig. 10A and B), here labeled cells were mostly scattered (Fig. 10E). Close inspection of the settling patterns suggested that these cells could have reached their positions in the medial and lateral hypothalamus according to different strategies. In the medial hypothalamus, labeled cells were usually radially arranged and in some cases their trail could be followed to the labeled neuroepithelium (Fig. 10E and G). In the lateral hypothalamus, however, labeled cells were not disposed in radial columns and lacked an obvious connection to the neuroepithelium, suggesting a tangential migration from caudal levels (Fig. 10E and F). Finally, another group without obvious relation to the neuroepithelium at this level was consistently found in the lateral hypothalamus, ventral to the cerebral peduncle (Fig. 10H). Our results are summarized in Fig. 11.

## Discussion

Because of the position of its rostral expression boundary, *Foxb1* lineage-labeling can detect tangential migrations from caudal levels into the rostral diencephalon and the telencephalon. One caveat is that inside the rostral diencephalon there is a source of *Foxb1*-lineage cells, the mammillary area, which contributes cells to more rostral regions (caudorostral intrahypothalamic migration). *Foxb1*-lineage mapping reveals overall cell migrations but is not appropriate for detailed mechanistic analysis of specific migrating cohorts whose settling point is known (see for instance Henderson *et al.*, 1999).

### Tangential migration in the hypothalamus

Hypothalamic migration is mostly radial (Altman & Bayer, 1986), which does not exclude tangential migration, as most of the forebrain shows a mixed pattern of radial and nonradial migration (Balaban *et al.*, 1988). Tangential migration uses axons as a substrate (axonophilic/neuronophilic migration; Rakic, 1990; Golden *et al.*, 1997), but neurons can migrate through a permissive corridor of membrane-attached molecules (Wichterle *et al.*, 2003) or even precede the axons (Lopez-Bendito *et al.*, 2006). Several functionally important neuronal groups enter the hypothalamus following nonradial trajectories. Gonadotrophin releasing hormone-expressing neurons migrate from nasal epithelium into hypothalamus following tangential axons (Muske & Moore, 1988; Schwanzel-Fukuda & Pfaff, 1989; Wray *et al.*, 1989) in a precisely regulated manner (reviewed in Schwarting *et al.*, 2007). A unique radial-to-tangential migration stream follows radial processes from the lateral ventricle neuroepithelium (telencephalon), entering the diencephalon tangentially to settle in the medial hypothalamus, ventral to the anterior commissure (Henderson *et al.*, 1999). This estrogen-controlled migration results in sexual dimorphism in the preoptic/anterior area (Wolfe *et al.*, 2005; Knoll *et al.*, 2007).

### Tangential migration of Foxb1-lineage cells into the lateral hypothalamus

Here we uncover a group of *Foxb1*-lineage cells extending from the prethalamus into a restricted lateral hypothalamic region as the result of tangential, axonophilic migration. Although we still do not have a full neurochemical characterization of hypothalamic *Foxb1*-lineage cells, we show that some *Foxb1*-lineage lateral hypothalamic neurons express specific lateral hypothalamic markers (hypocretin or MCH). Developmental, connectional and neurochemical heterogeneity of the MCH-expressing neurons has been reported (Brischoux *et al.*, 2001, 2002; Cvetkovic *et al.*, 2004), but to our knowledge the hypocretin/orexin-expressing population has until now been considered homogeneous (Amiot *et al.*, 2005).

Other descriptions of hypothalamic development contain data compatible with this migration. Expression of calretinin shows an early caudal diencephalic domain later extending into the lateral hypothalamus (Abbott & Jacobowitz, 1999), possibly representing a migration stream similar to the one we describe here. Fate-mapping studies of the avian neural plate can provide evidence of longitudinal cell migration. In chicken, tissue grafted in the vicinity of the thalamic eminence and prethalamus generates cells for the lateral hypothalamus (experiment QFM-38 in Cobos *et al.*, 2001) or dorsal hypothalamus and preoptic area (experiment R-173 in Garcia-Lopez *et al.*, 2004). While our mouse data agree in general with these results, it is not immediately obvious whether they describe comparable phenomena.

Additionally, the lateral hypothalamus (tuberal level) contains many labeled cells not radially arranged that could originate in the mammillary area then migrate caudorostrally. One example is the caudal-to-rostral migration from the mammillary body settling in a circumscribed area of the lateral hypothalamus (Fig. 7I). As these cells express *Foxb1* (Alvarez-Bolado *et al.*, 2000a), it is in principle possible that they do not migrate but appear to do so.

### Neuroepithelial migration into cortex

Part of the lateral hypothalamic neuroepithelium actually migrates from midbrain levels (Manning *et al.*, 2006). Tangential migration of neuroepithelial cells inside the diencephalon is a known but infrequent phenomenon (Arnold-Aldea & Cepko, 1996; Golden *et al.*, 1997). We show that a reduced number of neuroepithelial cells migrates early from caudal diencephalon into telencephalon (Fig. 4A–D), presumably originating the rare *Foxb1*-lineage cells that we have found in the cortex and which are often interneurons (coexpressing GAD). As far as we know, this would be the first report of neuroepithelial migration from diencephalon into cortex giving rise to interneurons. In the mouse, as a rule, cortical interneurons are generated in the basal ganglia (Marin & Rubenstein, 2003).

Additionally, we show what appears to be clonal expansion of the neuroepithelium in a very specific hypothalamic region giving rise to cells for the tuberal level.

### Different types of radial migration in the hypothalamus

Tuberal nuclei (ventromedial and arcuate) are generated locally by the neuroepithelium of the third ventricle (McClellan *et al.*, 2008). However, some radially migrating *Foxb1*-lineage cells seem to contribute to the arcuate, suggesting a contribution from ‘expanded’ neuroepithelium. Radial migration from neuroepithelium which has previously expanded tangentially can be considered a special case of the general radial pattern found in hypothalamus. Other specific hypothalamic subpopulations show characteristic variations on radial migration, for instance the parvicellular endocrine neurons migrate tangentially but do not follow the outside-in rule (Markakis & Swanson, 1997).

### Diencephalic migration into the telencephalon

No clearcut boundary separates diencephalon from telencephalon (Inoue *et al.*, 2000; Puelles *et al.*, 2000; Trujillo *et al.*, 2005), allowing for migration between them (Mitrofanis, 1994; Henderson *et al.*, 1999; Letinic & Rakic, 2001; Morante-Oria *et al.*, 2003). We have detected a novel migration stream from the dorsal diencephalon to the posterior septum and possibly the globus pallidus, as well as a migratory route from the caudal hypothalamus into piriform cortex and amygdala. Two caveats apply here. A subpopulation of piriform cortex neurons expresses *Foxb1* at a later stage of development (∼E14.5; Alvarez-Bolado *et al.*, 1999), so they may not be related to more caudal neuroepithelium by lineage. Besides, a reduced number of *Foxb1* lineage-labeled ventral midline cells reach the lamina terminalis and enter the basal ganglia (Fig. 3B and D), and some prethalamic neuroepithelial cells migrate into the telencephalon (Fig. 4A–D); therefore, some of the labeled cells in septum and pallidum could originate in migrated neuroepithelium (as opposed to tangentially migrating through the mantle).

### Foxb1 lineage in the ventral midline

The dorsal and ventral portions of the early neural tube meet at optic sulcus level, marking the ventral midline’s rostral end (Barth & Wilson, 1995; Shimamura *et al.*, 1995). At E8.5 and E9.5, the rostral end of the *Foxb1*-lineage domain is also at the optic level (as in zebrafish; Varga *et al.*, 1999). Later, the ventral midline *Foxb1* lineage extends briefly into dorsal levels (E11.5; Fig. 3B) to finally end at tuberal level (E12.5; Fig. 3D), due to differential growth of forebrain areas. In zebrafish and *Xenopus* the hypothalamus derives mostly from the ventral midline (Eagleson & Harris, 1990; Woo & Fraser, 1995; Varga *et al.*, 1999; Staudt & Houart, 2007). The present work and previous fate-mapping data (chicken, Cobos *et al.*, 2001; Fernandez-Garre *et al.*, 2002; Garcia-Lopez *et al.*, 2004; mouse, Inoue *et al.*, 2000) show that the hypothalamus of birds and mammals originates from more dorsal and lateral neuroepithelium as well.

## Abbreviations

3V: third ventricle
AHA: anterior hypothalamic area
AMY: amygdala
ARH: arcuate nucleus
CTX: cortex
DI: diencephalon
E: embryonic day
GAD: glutamic acid decarboxylase
GFAP: glial fibrillary acidic protein
GP: globus pallidus
hPLAP: human placental alkaline phosphatase
HYP: hypothalamus
ic: internal capsule
inf: infundibulum
ISH: *in situ* hybridization
LGd: lateral geniculate dorsal
LHA: lateral hypohalamic area
LM: lateral mammillary nucleus
LV: lateral ventricle
MAM: mammillary area
MBO: mammillary body
MCH: melanin concentrating hormone
os: optic sulcus
P: postnatal day
PBS: phosphate-buffered saline
PBT: phosphate-buffered Tween
PH: posterior hypothalamus
PIR: piriform cortex
PRO: preoptic area
PT: pretectum
PTh: prethalamus (ventral thalamus)
RT: room temperature
TG: tegmentum
Th: thalamus (dorsal thalamus)
TL: telencephalon
TUB: tuberal area
ZI: zona incerta

## References

[b1] Abbott LC, Jacobowitz DM (1999). Developmental expression of calretinin-immunoreactivity in the thalamic eminence of the fetal mouse. Int. J. Dev. Neurosci..

[b2] Altman J, Bayer SA (1986). The development of the rat hypothalamus. Adv. Anat. Embryol..

[b3] Alvarez-Bolado G, Swanson LW (1996). Developmental Brain Maps: The Structure of the Embryonic Rat Brain.

[b4] Alvarez-Bolado G, Rosenfeld MG, Swanson LW (1995). Model of forebrain regionalization based on spatiotemporal patterns of POU-III homeobox gene expression, birthdates, and morphological features. J. Comp. Neurol..

[b5] Alvarez-Bolado G, Cecconi F, Wehr R, Gruss P (1999). The fork head transcription factor Fkh5/Mf3 is a developmental marker gene for superior colliculus layers and derivatives of the hindbrain somatic afferent zone. Brain Res. Dev. Brain Res..

[b6] Alvarez-Bolado G, Zhou X, Cecconi F, Gruss P (2000a). Expression of Foxb1 reveals two strategies for the formation of nuclei in the developing ventral diencephalon. Dev. Neurosci..

[b7] Alvarez-Bolado G, Zhou X, Voss AK, Thomas T, Gruss P (2000b). Winged helix transcription factor Foxb1 is essential for access of mammillothalamic axons to the thalamus. Development.

[b8] Amiot C, Brischoux F, Colard C, La Roche A, Fellmann D, Risold PY (2005). Hypocretin/orexin-containing neurons are produced in one sharp peak in the developing ventral diencephalon. Eur. J. Neurosci..

[b9] Ang SL, Wierda A, Wong D, Stevens KA, Cascio S, Rossant J, Zaret KS (1993). The formation and maintenance of the definitive endoderm lineage in the mouse: involvement of HNF3/forkhead proteins. Development.

[b10] Arnold-Aldea SA, Cepko CL (1996). Dispersion patterns of clonally related cells during development of the hypothalamus. Dev. Biol..

[b11] Balaban E, Teillet MA, Le Douarin N (1988). Application of the quail-chick chimera system to the study of brain development and behavior. Science.

[b12] Barth KA, Wilson SW (1995). Expression of zebrafish nk2.2 is influenced by sonic hedgehog/vertebrate hedgehog-1 and demarcates a zone of neuronal differentiation in the embryonic forebrain. Development.

[b13] Brischoux F, Fellmann D, Risold PY (2001). Ontogenetic development of the diencephalic MCH neurons: a hypothalamic ‘MCH area’ hypothesis. Eur. J. Neurosci..

[b14] Brischoux F, Cvetkovic V, Griffond B, Fellmann D, Risold PY (2002). Time of genesis determines projection and neurokinin-3 expression patterns of diencephalic neurons containing melanin-concentrating hormone. Eur. J. Neurosci..

[b15] Caqueret A, Yang C, Duplan S, Boucher F, Michaud JL (2005). Looking for trouble: a search for developmental defects of the hypothalamus. Horm. Res..

[b16] Caqueret A, Boucher F, Michaud JL (2006). Laminar organization of the early developing anterior hypothalamus. Dev. Biol..

[b17] Chenn A, McConnell SK (1995). Cleavage orientation and the asymmetric inheritance of Notch1 immunoreactivity in mammalian neurogenesis. Cell.

[b18] Cobos I, Shimamura K, Rubenstein JL, Martinez S, Puelles L (2001). Fate map of the avian anterior forebrain at the four-somite stage, based on the analysis of quail-chick chimeras. Dev. Biol..

[b19] Cvetkovic V, Brischoux F, Jacquemard C, Fellmann D, Griffond B, Risold PY (2004). Characterization of subpopulations of neurons producing melanin-concentrating hormone in the rat ventral diencephalon. J. Neurochem..

[b20] Dymecki SM, Kim JC (2007). Molecular neuroanatomy’s “Three Gs”: a primer. Neuron.

[b21] Eagleson GW, Harris WA (1990). Mapping of the presumptive brain regions in the neural plate of *Xenopus laevis*. J. Neurobiol..

[b22] Fernandez-Garre P, Rodriguez-Gallardo L, Gallego-Diaz V, Alvarez IS, Puelles L (2002). Fate map of the chicken neural plate at stage 4. Development.

[b23] Fields-Berry SC, Halliday AL, Cepko CL (1992). A recombinant retrovirus encoding alkaline phosphatase confirms clonal boundary assignment in lineage analysis of murine retina. Proc. Natl Acad. Sci. USA.

[b24] Fishell G, Mason CA, Hatten ME (1993). Dispersion of neural progenitors within the germinal zones of the forebrain. Nature.

[b25] Garcia-Lopez R, Vieira C, Echevarria D, Martinez S (2004). Fate map of the diencephalon and the zona limitans at the 10-somites stage in chick embryos. Dev. Biol..

[b26] Golden JA, Cepko CL (1996). Clones in the chick diencephalon contain multiple cell types and siblings are widely dispersed. Development.

[b27] Golden JA, Zitz JC, McFadden K, Cepko CL (1997). Cell migration in the developing chick diencephalon. Development.

[b28] Gustincich S, Feigenspan A, Wu DK, Koopman LJ, Raviola E (1997). Control of dopamine release in the retina: a transgenic approach to neural networks. Neuron.

[b29] Henderson RG, Brown AE, Tobet SA (1999). Sex differences in cell migration in the preoptic area/anterior hypothalamus of mice. J. Neurobiol..

[b30] Inoue T, Nakamura S, Osumi N (2000). Fate mapping of the mouse prosencephalic neural plate. Dev. Biol..

[b31] Joyner AL, Zervas M (2006). Genetic inducible fate mapping in mouse: establishing genetic lineages and defining genetic neuroanatomy in the nervous system. Dev. Dyn..

[b32] Kaestner KH, Schutz G, Monaghan AP (1996). Expression of the winged helix genes fkh-4 and fkh-5 defines domains in the central nervous system. Mech. Dev..

[b33] Kloetzli JM, Fontaine-Glover IA, Brown ER, Kuo M, Labosky PA (2001). The winged helix gene, Foxb1, controls development of mammary glands and regions of the CNS that regulate the milk-ejection reflex. Genesis.

[b34] Knoll JG, Wolfe CA, Tobet SA (2007). Estrogen modulates neuronal movements within the developing preoptic area-anterior hypothalamus. Eur. J. Neurosci..

[b35] Koenen M, Ruther U, Muller-Hill B (1982). Immunoenzymatic detection of expressed gene fragments cloned in the lac Z gene of E. coli. EMBO J..

[b36] Labosky PA, Winnier GE, Jetton TL, Hargett L, Ryan AK, Rosenfeld MG, Parlow AF, Hogan BL (1997). The winged helix gene, Mf3, is required for normal development of the diencephalon and midbrain, postnatal growth and the milk-ejection reflex. Development.

[b37] Leighton PA, Mitchell KJ, Goodrich LV, Lu X, Pinson K, Scherz P, Skarnes WC, Tessier-Lavigne M (2001). Defining brain wiring patterns and mechanisms through gene trapping in mice. Nature.

[b38] Letinic K, Rakic P (2001). Telencephalic origin of human thalamic GABAergic neurons. Nat. Neurosci..

[b39] Lobe CG, Koop KE, Kreppner W, Lomeli H, Gertsenstein M, Nagy A (1999). Z/AP, a double reporter for cre-mediated recombination. Dev. Biol..

[b40] Lopez-Bendito G, Cautinat A, Sanchez JA, Bielle F, Flames N, Garratt AN, Talmage DA, Role LW, Charnay P, Marin O, Garel S (2006). Tangential neuronal migration controls axon guidance: a role for neuregulin-1 in thalamocortical axon navigation. Cell.

[b41] Louvi A, Yoshida M, Grove EA (2007). The derivatives of the Wnt3a lineage in the central nervous system. J. Comp. Neurol..

[b42] Manning L, Ohyama K, Saeger B, Hatano O, Wilson SA, Logan M, Placzek M (2006). Regional morphogenesis in the hypothalamus: a BMP-Tbx2 pathway coordinates fate and proliferation through Shh downregulation. Dev. Cell.

[b43] Marin O, Rubenstein JL (2003). Cell migration in the forebrain. Annu. Rev. Neurosci..

[b44] Markakis EA, Swanson LW (1997). Spatiotemporal patterns of secretomotor neuron generation in the parvicellular neuroendocrine system. Brain Res. Brain Res. Rev..

[b45] McClellan KM, Calver AR, Tobet SA (2008). GABAB receptors role in cell migration and positioning within the ventromedial nucleus of the hypothalamus. Neuroscience.

[b46] Michaud JL (2001). The developmental program of the hypothalamus and its disorders. Clin. Genet..

[b47] Mitrofanis J (1994). Development of the thalamic reticular nucleus in ferrets with special reference to the perigeniculate and perireticular cell groups. Eur. J. Neurosci..

[b48] Morante-Oria J, Carleton A, Ortino B, Kremer EJ, Fairen A, Lledo PM (2003). Subpallial origin of a population of projecting pioneer neurons during corticogenesis. Proc. Natl Acad. Sci. USA.

[b49] Muske LE, Moore FL (1988). The nervus terminalis in amphibians: anatomy, chemistry and relationship with the hypothalamic gonadotropin-releasing hormone system. Brain Behav. Evol..

[b50] Paxinos G, Franklin KBJ (2001). The Mouse Brain in Stereotaxic Coordinates.

[b51] Puelles L, Rubenstein JL (2003). Forebrain gene expression domains and the evolving prosomeric model. Trends Neurosci..

[b52] Puelles L, Kuwana E, Puelles E, Bulfone A, Shimamura K, Keleher J, Smiga S, Rubenstein JL (2000). Pallial and subpallial derivatives in the embryonic chick and mouse telencephalon, traced by the expression of the genes Dlx-2, Emx-1, Nkx-2.1, Pax-6, and Tbr-1. J. Comp. Neurol..

[b53] Rakic P (1988). Specification of cerebral cortical areas. Science.

[b54] Rakic P (1990). Principles of neural cell migration. Experientia.

[b55] Schwanzel-Fukuda M, Pfaff DW (1989). Origin of luteinizing hormone-releasing hormone neurons. Nature.

[b56] Schwarting GA, Wierman ME, Tobet SA (2007). Gonadotropin-releasing hormone neuronal migration. Semin. Reprod. Med..

[b57] Shimamura K, Hartigan DJ, Martinez S, Puelles L, Rubenstein JL (1995). Longitudinal organization of the anterior neural plate and neural tube. Development.

[b58] Sillitoe RV, Joyner AL (2007). Morphology, molecular codes, and circuitry produce the three-dimensional complexity of the cerebellum. Annu. Rev. Cell Dev. Biol..

[b59] Simerly RB, Paxinos G (2004). Anatomical substrates of hypothalamic integration. The Rat Nervous System.

[b60] Soriano P (1999). Generalized lacZ expression with the ROSA26 Cre reporter strain. Nat. Genet..

[b61] Staudt N, Houart C (2007). The prethalamus is established during gastrulation and influences diencephalic regionalization. PLoS Biol..

[b62] Swanson LW, Björlund A, Hökfelt T, Swanson LW (1987). The hypothalamus. Handbook of Chemical Neuroanatomy.

[b63] Tobet SA (2002). Genes controlling hypothalamic development and sexual differentiation. Eur. J. Neurosci..

[b64] Trujillo CM, Alonso A, Delgado AC, Damas C (2005). The rostral and caudal boundaries of the diencephalon. Brain Res. Brain Res. Rev..

[b65] Varga ZM, Wegner J, Westerfield M (1999). Anterior movement of ventral diencephalic precursors separates the primordial eye field in the neural plate and requires cyclops. Development.

[b66] Wehr R, Mansouri A, de Maeyer T, Gruss P (1997). Fkh5-deficient mice show dysgenesis in the caudal midbrain and hypothalamic mammillary body. Development.

[b67] Wichterle H, Alvarez-Dolado M, Erskine L, Alvarez-Buylla A (2003). Permissive corridor and diffusible gradients direct medial ganglionic eminence cell migration to the neocortex. Proc. Natl Acad. Sci. USA.

[b68] Wilkinson DG (1992). In Situ Hybridization: A Practical Approach.

[b69] Wolfe CA, Van Doren M, Walker HJ, Seney ML, McClellan KM, Tobet SA (2005). Sex differences in the location of immunochemically defined cell populations in the mouse preoptic area/anterior hypothalamus. Brain Res. Dev. Brain Res..

[b70] Woo K, Fraser SE (1995). Order and coherence in the fate map of the zebrafish nervous system. Development.

[b71] Wray S, Grant P, Gainer H (1989). Evidence that cells expressing luteinizing hormone-releasing hormone mRNA in the mouse are derived from progenitor cells in the olfactory placode. Proc. Natl Acad. Sci. USA.

[b72] Zervas M, Blaess S, Joyner AL (2005). Classical embryological studies and modern genetic analysis of midbrain and cerebellum development. Curr. Top. Dev. Biol..

[b73] Zhao T, Zhou X, Szabo N, Leitges M, Alvarez-Bolado G (2007). Foxb1-driven Cre expression in somites and the neuroepithelium of diencephalon, brainstem, and spinal cord. Genesis.

